# Gene expression in salivary glands: effects of diet and mouse chromosome 17 locus regulating macronutrient intake

**DOI:** 10.14814/phy2.12311

**Published:** 2015-02-23

**Authors:** Jacob Simon, Lisa M DiCarlo, Claudia Kruger, William D Johnson, Claudia Kappen, Brenda K Richards

**Affiliations:** 1 Genetics of Eating Behavior Laboratory, Pennington Biomedical Research Center, Louisiana State University SystemBaton Rouge, Louisiana; 2 Department of Developmental Biology, Pennington Biomedical Research Center, Louisiana State University SystemBaton Rouge, Louisiana; 3 Department of Biostatistics, Pennington Biomedical Research Center, Louisiana State University SystemBaton Rouge, Louisiana

**Keywords:** Gene expression, hyaluronan, salivary gland, subcongenic mice

## Abstract

*Dcpp2*, *Prrt1,* and *Has1* are plausible candidate genes for the *Mnic1* (macronutrient intake-carbohydrate) locus on mouse chromosome 17, based on their map positions and sequence variants, documented expression in salivary glands, and the important role of saliva in oral food processing and taste. We investigated the effects of genotype and diet on gene expression in salivary glands (parotid, submandibular, sublingual) of carbohydrate-preferring, C57BL6J.CAST/EiJ-17.1 subcongenic mice compared to fat-preferring wild-type C57BL/6J. To achieve accurate normalization of real-time quantitative RT-PCR data, we evaluated multiple reference genes to identify the most stably expressed control genes in salivary gland tissues, and then used geometric averaging to produce a reliable normalization factor. Gene expression was measured in mice fed different diets: (1) rodent chow, (2) macronutrient selection diets, (3) high-fat diet, and (4) low-fat diet. In addition, we measured salivary hyaluronan concentrations. All three genes showed strain differences in expression, in at least one major salivary gland, and diet effects were observed in two glands. *Dcpp2* expression was limited primarily to sublingual gland, and strongly decreased in B6.CAST-17.1 subcongenic mice compared to wild-type B6, regardless of diet. In contrast, both genotype and diet affected *Prrt1* and *Has1* expression, in a gland-specific manner, for example, *Prrt1* expression in the parotid gland alone was strongly reduced in both mouse strains when fed macronutrient selection diet compared to chow. Notably, we discovered an association between diet composition and salivary hyaluronan content. These results demonstrate robust effects of genetic background and diet composition on candidate gene expression in mouse salivary glands.

## Introduction

A person's choice of carbohydrate- versus fat-rich foods is a complex trait that depends on both genetic and environmental factors (Comuzzie 2004; Grimm and Steinle 2011). In fact, there is growing evidence for a strong genetic influence on appetite traits in both children and adults (Cai et al. 2006; Rankinen and Bouchard 2006; Choquette et al. 2008; Reed 2010), yet the specific genes responsible for the quantitative variation in macronutrient preference are virtually unknown. Understanding the processes by which food choices are made, and ensuring that those choices are healthy, is important for addressing the epidemics of obesity, diabetes, and cardiovascular disease. We and others have shown that mice, similar to people, vary in the amount of carbohydrate and fat they eat when selecting among food choices (Reed et al. 1997; Smith et al. 1997, 1999, 2000). Mice are excellent models for genetic mapping and follow-up studies, for example, interval-specific strains can be bred to isolate and test the phenotypic effects of loci associated with complex traits.

In previous work, we discovered a QTL region on mouse chromosome 17 that regulates an animal's choice to eat more carbohydrate- rather than fat-rich food, and was therefore named *Mnic1* (macronutrient intake-carbohydrate) (Smith Richards et al. 2002; Kumar et al. 2008). We experimentally confirmed that this genetic interval contains a gene or genes responsible for carbohydrate preference (Kumar et al. 2010) by developing a subcongenic strain that differs from the control strain only in the *Mnic1* region. B6.CAST-^*D17Mit19-D17Mit50*^ (B6.CAST-17.1) mice possess a ∼40.1 Mb region of CAST DNA on mouse chromosome 17, on an otherwise B6 genome. When tested in a macronutrient selection paradigm (carbohydrate- vs. fat-rich diets), the B6.CAST-17.1 subcongenic mice selected/consumed ∼30% more calories per body weight from the carbohydrate-rich diet, compared to wild-type B6 littermates (Kumar et al. 2010).

Saliva plays an important role in oral food processing. In particular, saliva initiates digestion, for example, 80–90% of the daily saliva output is produced in response to stimuli such as taste, smell, and mastication (Melvin et al. 2005). Saliva may also modulate taste stimuli by solubilizing and transporting taste molecules (Spielman 1990; Salles et al. 2011). Thus, saliva could influence diet choice by affecting the perception of texture or flavor. Saliva contains electrolytes and more than 1000 different proteins whose functions are yet unknown (Gonzalez-Begne et al. 2011), and could have effects on food intake or food choice. Conversely, the secretion or function, for example, binding activities, of salivary proteins may be modified by diet composition (Lamy et al. 2010). Because of the implications of these relationships for oral health, nutrition, and commercial applications, it is important to understand the interaction of genetic and diet factors in the expression of salivary proteins (Torregrossa et al. 2014).

To identify potential candidate genes for macronutrient selection, we examined known genes located within the *Mnic1* 95% confidence interval, under large inflections in the LOD score. We noted that several of these genes were expressed in the salivary gland: *Dcpp2* (demilune cell and parotid protein 2), *Prrt1* (proline-rich transmembrane protein 1), and *Has1* (hyaluronan synthase 1), although their functional relevance remains to be fully elucidated. Sequence-based analysis (http://phenome.jax.org/) revealed SNPs (single nucleotide polymorphisms) in *Has1* and *Dcpp2* producing nonsynonymous changes in the coding sequence between B6 and CAST, which may alter protein function. Although the *Dcpp* gene family consists of three closely linked genes on mouse chromosome 17 (Mullins et al. 2006), only *Dcpp2,* and not *Dcpp1* or *Dcpp3*, has a documented missense mutation between strains (CAST/EiJ, C57BL/6J) in our genetic model. We undertook these studies based on the assumption that a candidate QTL gene must be expressed in a trait-relevant tissue (e.g., salivary gland) and exhibit expression variation relative to differing conditions (e.g., diet) that are related to the trait of interest (macronutrient diet selection) (Flint et al. 2005; Arbilly et al. 2006). We viewed a genotype-by-diet interaction affecting gene expression as strong evidence that a gene(s) may contribute to the QTL. Accordingly, in this study, we measured the effects of genotype and diet type on the expression of *Dcpp2*, *Prrt1,* and *Has1* in mouse salivary glands (parotid, submandibular, sublingual) in carbohydrate-preferring B6.CAST-17.1 subcongenic compared to fat-preferring wild-type mice. We also measured salivary hyaluronic acid (HA) concentrations under different diet conditions.

## Methods

### Ethics statement

All experimental protocols were approved by the Pennington Biomedical Research Center (PBRC) Institutional Animal Care and Use Committee and therefore were performed in accordance with the Public Health Service Policy on Humane Care and Use of Laboratory Animals; the Animal Welfare Act and Regulations*;* and the Guide for the Care and Use of Laboratory Animals. PBRC is accredited by the Association for the Assessment and Accreditation of Laboratory Animal Care International (AAALAC, Int., Frederick, MD).

### Animals and experimental design

Phenotypic and genomic data were generated from B6.CAST-17.1 subcongenic and B6 wild-type mice. The development of the B6.CAST-^*D17Mit19-D17Mit50*^ subcongenic and wild-type strains has been described previously (Kumar et al. 2010). Recently, this chromosome 17 subcongenic interval was more precisely defined as a 42.5 Mb interval bounded by SNP markers *rs49640908* (proximal end of segment) and *rs48762654* (distal) (Gularte-Merida et al. 2014). Experimental animals were bred in our local colony at the PBRC. Animals were singly housed in polycarbonate cages with sterilized corncob bedding at 22–23°C and kept on a 12:12 h light/dark cycle. Mice were provided with tap water and no. 5001 chow (LabDiets, Richmond, IN) until initiation of experimental diets.

### Experimental diets

Complete details of the phenotyping procedures have been described previously (Smith Richards et al. 2002) and the experimental diet composition is listed in Table1. Briefly, macronutrient diet selection phenotypes were assessed by providing mice with a choice between fat/protein (F/P) and carbohydrate/protein (C/P) diet mixtures, equivalent for protein (22% of energy) and with the balance of calories contributed by either fat or carbohydrate (78%). Both diets contained casein (protein), minerals, and vitamins. The C/P diet was composed of corn starch and powdered sucrose, whereas the F/P diet contained vegetable shortening from one of two sources, according to availability. Each diet was presented in a custom two oz. glass jar with stainless steel lid (Unifab, Kalamazoo, MI); food intake and all spillage were measured daily to the nearest 0.1 g. The open source high- (HF) and low-fat (LF) experimental diets were equivalent for protein (16.4% of energy) with the balance of calories contributed by 58% fat and 25.5% carbohydrate in D12331 and by 10.5% fat and 73.1% carbohydrate in D12329 (Research Diets, Inc., New Brunswick, NJ). The no. 5001 rodent chow contains 28.5% protein, 13.5% fat and 58% carbohydrate by energy (LabDiets).

**Table 1:** Composition of macronutrient-rich diets^1^

2-choice	Carbohydrate/protein	Fat/protein
Corn starch	49.62	0.00
Powdered sugar	21.24	0.00
Casein	19.88	32.77
DL-methionine	0.29	0.49
Vegetable shortening	0.00	51.93
AIN-76A vitamin mix^2^	1.00	1.53
AIN-76A mineral mix^2^	3.20	5.33
Choline chloride	0.18	0.31
Cellulose (alphacel)	4.92	7.62
Energy density (kcal/g)	3.61	5.96

1 Ingredients expressed as percent by weight.

2 The vitamin and mineral mixes contain 97% and 12% sucrose, respectively.

### Saliva induction and collection

Approximately 2 h prior to the procedure, the individually housed mice were weighed and food (not water) was removed from their cage. Prior to saliva collection, the animals were administered light anesthesia using rodent cocktail (80 mg/mL ketamine/1.6 mg/mL acepromazine/4 mg/mL xylazine) diluted 1:5 with sterile water and administered at a dose of 0.125 mL/20 g body weight. For terminal procedures involving tissue collection, deep anesthesia was induced using isoflurane/O2 inhalation. Salivation then was induced by the subcutaneous injection of epinephrine (4.0–6.0 mg/kg) (Wallace and Partlow 1976). Approximately 5 min later, a micropipette tip was used to collect saliva from the oral cavity over a ∼10 min period and the sample was frozen at −20°C. In Experiment 1, mice were recovered from light anesthesia and returned to their home cage. In Experiment 2, saliva was collected from deeply anesthetized mice prior to euthanasia. All samples were collected during the middle of the light period.

### Outline of experiments

#### Effects of diet and genotype on salivary hyaluronic acid levels

In two experiments, we collected saliva from male, 12–16 week old mice for the purpose of measuring hyaluronic acid (HA). In Experiment 1, a within-subjects design was used to assess the induction of HA 48 h after initiation of macronutrient selection diets (fat- vs. carbohydrate-rich diets). Saliva was collected from lightly anesthetized subcongenic (*n* = 9) and wild-type mice (*n* = 9), first while they were fed rodent chow, and then again after 2 days of macronutrient selection diet (see Table1). In Experiment 2, saliva was collected from deeply anesthetized subcongenic (*n* = 10) and wild-type (*n* = 10) 48 h after initiation of high- (HF) or low-fat (LF) diet. Following saliva collection, salivary glands were dissected out and quickly frozen in liquid nitrogen. In both experiments, food intake was measured daily.

#### Effects of diet and genotype on gene expression in salivary glands

Two experiments were conducted in which salivary glands were harvested from 11- to 12-week-old male mice, fed either high- or low-fat diet for 2 day (Experiment 3); or chow or 2-choice macronutrient selection diet for 2 day (Experiment 4). Tissues were harvested from deeply anesthetized animals in a terminal procedure. The same animals used to collect saliva samples in Experiment 2 were used to obtain the tissues analyzed in Experiment 3.

### Tissue collection and preparation of RNA for gene expression analyses

The right lobe of each salivary gland (sublingual, submandibular, parotid), minus any salivary lymph nodes, was dissected (Amano et al. 2012), frozen in liquid nitrogen, and stored at −80°C. Total RNA from sublingual and submandibular glands was isolated using the reagent TRIzol (Life Technologies Corporation, Carlsbad, CA) and from parotid tissue using the Qiagen RNeasy Mini Kit column extraction kit (Qiagen, Germantown, MD). RNA was quantified on a NanoDrop ND-1000 spectrophotometer (Thermo Scientific Inc., Wilmington, DE), and stored at −80°C until further analysis. Complementary DNA was obtained by reverse transcription (Reverse Transcriptase cDNA Synthesis kit (Qiagen) of at least 1 µg of RNA of each sample, according to manufacturer's instructions.

### Primers and real-time quantitative RT-PCR

Gene sequences were obtained from the National Center for Biotechnology Information (NCBI) using GenBank Release 190.0 in June 2012 (www.ncbi.nlm.nih.gov/nucleotide/). Primers were designed using Primer Express Software v3.0 (Life Technologies). The locations and sequences of primers are listed in Table2. An examination of the amplification efficiencies (AE) for all three primer sets, revealed equivalent AEs in both strains, indicating that the chosen primers detected/amplified the C57BL/6J and CAST/EiJ cDNAs with equal efficiencies (Table3). When possible, the primers were positioned to span exon–exon junctions so that amplification of potentially contaminating genomic DNA would be excluded. Gene expression was evaluated using the ABI Prism 7900HT Sequence Detection System (Life Technologies). Each PCR reaction (10 µL) was performed on 3 ng of template cDNA, 100 nmol/L of each primer, and SYBR® Green PCR Master Mix (Applied Biosystems, Carlsbad, CA). The PCR reactions consisted of a denaturation step of 15 sec at 95°C, annealing for 2 min at 50°C, and extension for 1 min at 60°C, for a total of 40 cycles. Each cDNA sample was assayed in triplicate, and the cycle number at first detection of signal above threshold (Ct) was determined. A nontemplate control was included for each primer set to confirm the absence of genomic DNA and to check for primer-dimer or contamination in the reactions, respectively. To ensure that only a single product was amplified, a melting curve analysis was performed. Analysis was performed using the SDS Software v2.4 for the 7900HT (Life Technologies).

**Table 2:** Primers used for the real-time quantitative RT-PCR assays

Gene symbol	Accession #	Forward primer sequence	Position	Reverse primer sequence	Exon-exon boundary	AE
Actb	NM_007393	5′-AGCCTTCCTTCTTGGGTATGG-3′	788–862	5′-CAACGTCACACTTCATGATGGA-3′	Yes	1.89
B2m	NM_009735	5′-TCCAGAAAACCCCTCAAATTCA-3′	62–127	5′-GTATGTTCGGCTTCCCATTCTC-3′	Yes	1.95
Dcpp2	NM_001039238	5′-CCGGGTATTTCTTCAGGCTAGA-3′	144–209	5′-CCGTCCTCGTTGTCATTATAGTTG-3′	Yes	1.99
Gapdh	AK144690	5′-TGTGTCCGTCGTGGATCTGA-3′	711–787	5′-CCTGCTTCACCACCTTCTTGA-3′	Yes	1.93
Has1	NM_008215	5′-TCAGGGAGTGGGATTGTAGGA-3′	1980–2040	5′-AAATAGCAACAGGGAGAAAATGGA-3′	No	1.86
Hprtl	NM_013556	5′-TGACACTGGCAAAACAATGCA-3′	711–774	5′-GGTCCTTTTCACCAGCAAGCT-3′	No	1.84
Pole4	NM_025882	5′-CGGGACAGGAAGCCATCTT-3′	269–348	5′-AGCAGTAGGCATCTTTTGCGATA-3′	Yes	1.88
Ppia	AK151583	5′-AGGGTTCCTCCTTTCACAGAATTA-3′	146–219	5′-AGTGCCATTATGGCGTGTAAA-3′	Yes	1.99
Ppib	NM_011149	5′-GGTGGAGAGCACCAAGACAGA-3′	543–609	5′-GCCGGAGTCGACAATGATG-3′	No	1.84
Prrt1	NM_030890	5′-GTCCGCCACACGACTACATG-3′	653–789	5′-GATCTCGGCAGACACCAAATC-3′	Yes	1.86
Rpl27a	NM_011975	5′-AAAGCCGTCATCGTGAAGAAC-3′	64–164	5′-GCTGTCACTTTCCGGGGATAG-3′	Yes	1.93
Sdha	NM_023281	5′-CGGCTTTCACTTCTCTGTTGGT-3′	93–168	5′-TGGGTATTGAGTAGAAATTGCATCTG-3′	Yes	1.89

Primer sequences and positions for the reference sequence are given. When possible, primers were designed to span exon–exon junctions to avoid amplification of contaminating genomic DNA. Real-time qRT-PCR amplification efficiencies (AE) were calculated from the actual PCR runs.

**Table 3:** Amplification efficiencies (AE) for primer sets

Gene symbol	Genotype	AE ± SD
Dcpp2	CON	1.99 ± 0.18
	WT	1.98 ± 0.22
Prrt1	CON	1.91 ± 0.14
	WT	1.86 ± 0.16
Has1	CON	1.89 ± 0.17
	WT	1.86 ± 0.16

CON, subcongenic; WT, wild type; SD, standard deviation.

**Figure 1:**
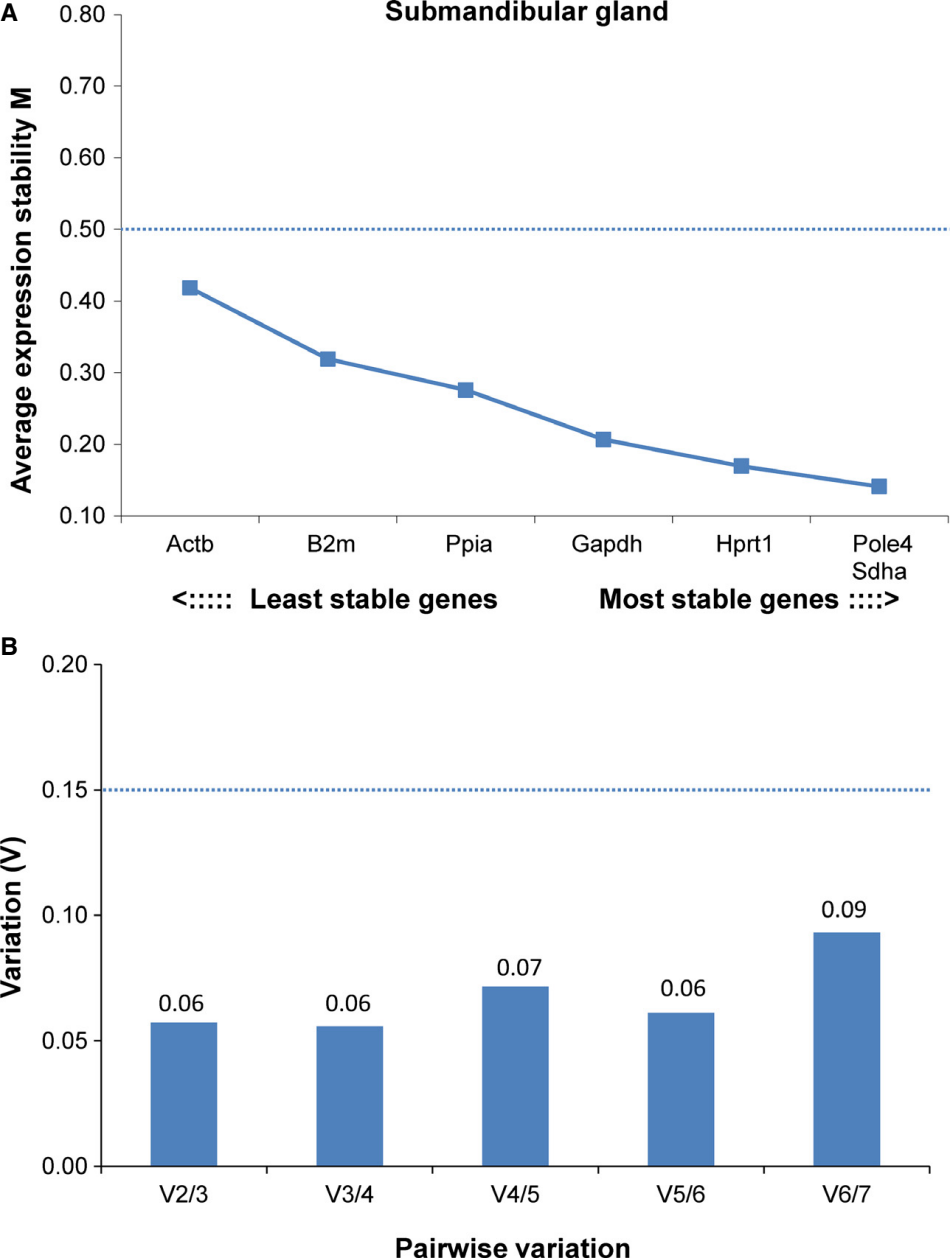
Gene expression stability and pairwise variation of the candidate reference genes for accurate normalization in submandibular gland. (A) The chart generated by geNorm indicates the average expression stability value (*M*) of reference genes at each step during stepwise exclusion of the least stable expressed reference gene. Starting from the least stable gene at the left, the genes are ranked according to increasing expression stability, ending with the two most stable genes on the right. In this analysis, *Pole4* and *Sdha* were the two most stable genes. (B) The second chart then guides the optimal number of reference genes. The process of determining “pairwise variation *V*” begins with the two most stably expressed genes on the left, followed by the inclusion of a 3rd, 4th, 5th gene, etc. moving to the right. A *V* score of below 0.15 (marked with a dashed line) is considered ideal, and use of the two most stable genes alone (*Pole4, Sdha*) was sufficient to obtain a *V* score of 0.06 which was not improved by the addition of a third gene (*V*3/4 = 0.06).

**Figure 2:**
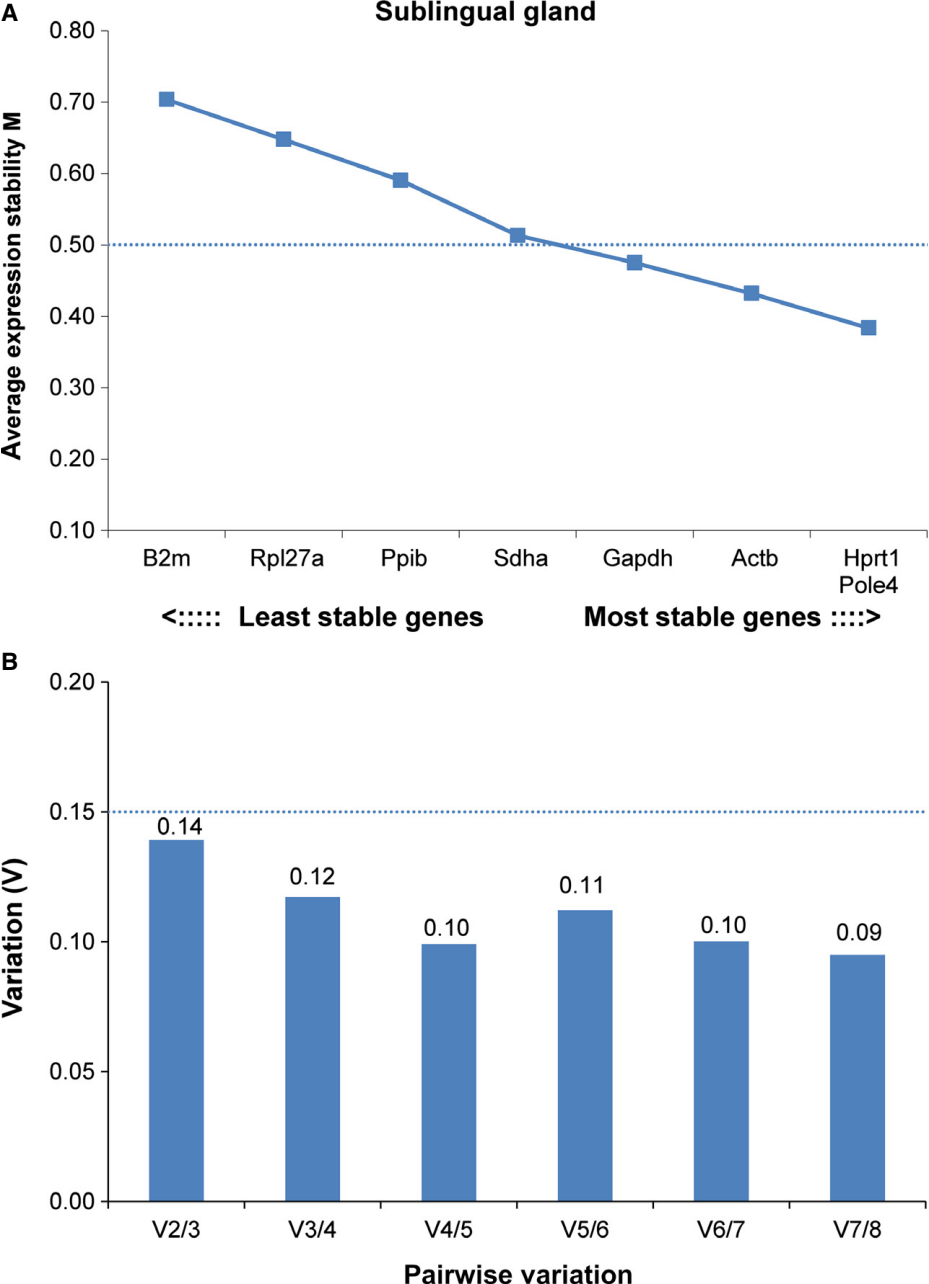
Gene expression stability and pairwise variation of the candidate reference genes for accurate normalization in sublingual gland. (A) In this analysis, *Hprt1* and *Pole4* were the two most stable genes, followed by *Actb*. (B) Although using the two most stable genes was sufficient to obtain a V score of 0.14, which is less than the cut-off of 0.15, the downward trend of *V* values suggested that using the three best references genes would be more optimal (3/4 = 0.12).

The qRT-PCR data were normalized using multiple reference genes, based on the results of our validation studies (see Results and Figs.1, 2). A geometric mean of at least two reference genes was calculated for each sample and used as the normalization factor. Using a geometric mean, instead of the arithmetic mean, is a better control for possible outlier values and differences in abundance between genes (Vandesompele et al. 2002). Triplicate measures for each sample were averaged, and the value obtained for each gene was then normalized to measurements of reference gene cDNA in the same sample, by applying the formula: 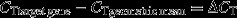. The target gene *C*_T_ values were normalized to the geometric mean of *Sdha* and *Pole4* for the submandibular gland; to *Hprt1*, *Pole4*, and *Actb* for the sublingual gland; and to *Hprt1*, *Pole4,* and *Sdha* for the parotid. All assays were performed on at least six samples. Statistical tests were carried out using the Δ*C*_T_ values. Comparison of subcongenic samples to those for wild-type B6 controls was accomplished using a second subtraction: Δ*C*_Tsubcongenic_ − Δ*C*_Twildtype_ = ΔΔ*C*_T_ values. To estimate the relative fold change (FC) in expression level, for comparison of selected samples, we used the equation: 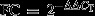, assuming an amplification efficiency of 2 (Schmittgen and Livak 2008). In this way, the FC data were interpreted as gene expression in subcongenic mice relative to the wild-type mice.

### Saliva sample preparation and ELISA for hyaluronic acid

Saliva samples were collected from mice and stored at −20°C until further processing. On the day that the samples were assayed for hyaluronic acid (HA), they were thawed at room temperature, mixed using a vortex mixer, and centrifuged at 3000 rpm (1500 *g*) for ∼15 min. to remove any sediment (www.salimetrics.com). The clear supernatant for each sample was then removed and diluted 1:10 in water. HA is a high molecular weight anionic polysaccharide (1000–10,000 kD) composed of repeating disaccharides of *β*(1-4)glucuronic acid and *β*(1-3)N-acetylglucosamine. HA concentrations in individual saliva samples were determined using an ELISA (Biotech Trading Partners, Encinitas, CA), which detects HA using a highly purified hyaluronic acid-binding protein (HABP) molecule isolated from bovine cartilage. The lower limit of detection is 10 ng/mL. Total protein concentrations were determined using a Pierce BCA Protein Determination Kit (Thermo Scientific, Waltham, MA) and final HA concentrations were expressed per ng of total protein. The PRRT1 and DCPP2 proteins were not explored because antibodies for these gene products were not available at the time these studies were conducted.

### Statistical analyses

The qRT-PCR data were analyzed using an unpaired two-tailed *t*-test with assumption of unequal variance (Microsoft Excel Analysis Pack). The response variables, that is, normalized *C*_T_ values, were analyzed using a two-way ANOVA with genotype and diet as factors. Bonferroni was used to correct for multiple comparisons. The hyaluronan data were analyzed using SAS version 9.4 (SAS Institute, Inc., Cary, NC). The dependent variable (HLA/total protein) was analyzed using ANCOVA with genotype and proportional fat intake as fixed effects. All values are shown as means ± SD unless otherwise noted. The threshold for significance was set at *P* < 0.05.

## Results

### geNorm analysis identified suitable reference genes for salivary gland

It is standard practice for mRNA levels of the target or gene-of-interest to be normalized to endogenous controls referred to as reference genes. Ideally, the reference gene should be expressed at a similar level as the genes of interest and exhibit low inter-animal variability. In preliminary studies, we observed that expression levels of commonly used reference genes, such as *Ppia*, differed between our strains when normalized to total RNA in the sample, and therefore were not suitable for use. We therefore chose to normalize our real-time quantitative RT-PCR data by geometric averaging of multiple internal control genes. The highly cited geNorm algorithm was used to identify the most stably expressed genes in our system, from a pool of candidates. The geNorm output consists of an *M* value, defined as the average pairwise variation of a particular gene with all other control genes within a given set of samples (Vandesompele et al. 2002), assuming that the control genes are not coregulated. The program provides for the elimination of the worst-scoring reference gene, that is, the one with the highest *M* value, and recalculates the *M* value for the remaining genes. Genes with the lowest *M* values have the most stable expression. In addition, geNorm determines the optimal number of control genes for normalization based on a pairwise variation (*V*_*n*/*n *+ 1_) analysis (Vandesompele et al. 2002).

A total of 10 internal control genes were selected for analysis: *β*-actin (*Actb*), beta-2 microglobulin (*B2m*), glyceraldehyde-3-phosphate dehydrogenase (*Gapdh*), hypoxanthine guanine phosphoribosyl transferase (*Hprt1*), polymerase (DNA-directed), epsilon 4 (p12 subunit) (*Pole4*), peptidylprolyl isomerase A (*Ppia*), peptidylprolyl isomerase B (*Ppib*), ribosomal protein L27A (*Rpl27a*), succinate dehydrogenase complex, subunit A, and flavoprotein (Fp) (*Sdha*) as shown in Table2. The amplification efficiencies of all qPCR reactions were ≥1.8 (Table2).

Expression data from a panel of eight genes in sublingual and seven genes in submandibular gland were analyzed using geNorm (Vandesompele et al. 2002). In submandibular gland, all seven genes tested exhibited high expression stability with *M* values below 0.5 (Fig.1). In sublingual gland, *Gapdh*, *Actb*, *Hprt1,* and *Pole4* exhibited high expression stability with *M* values below 0.5, whereas four other genes (*B2m*, *Rpl27a*, *Ppib*, *Sdha*) showed *M* values between 0.5 and 0.7 (Fig.2). *Pole4* proved to be the most stable gene in both glands. For each gland, we combined the two or three most stably expressed genes to provide optimal normalization, as defined by pairwise variation below the cut-off value of *V* = 0.15 (Vandesompele et al. 2002) (Figs.1B, 2B). For submandibular gland, the *V*2/3 value was considered optimal because including a third reference gene did not significantly change the normalization factor (Fig.1B); for this reason, *Pole4* and *Sdha* were used as control genes for the submandibular gland. For sublingual gland, the *V*3/4 value of 0.11 was considered as sufficient, despite the fact that the optimal number of reference genes in this analysis would be four, according to the *V*4/5 value of 0.09 (Fig.2A). Based on these results, *Actb*, *Hprt1,* and *Pole4* were used to normalize expression data from the sublingual gland. Three of the most stably expressed genes identified in these two glands (*Hprt1*, *Pole4, Sdha*) were used as controls for qRT-PCR analysis in the parotid gland.

### *Dcpp2* expression in the sublingual gland was altered by genotype

**Figure 3:**
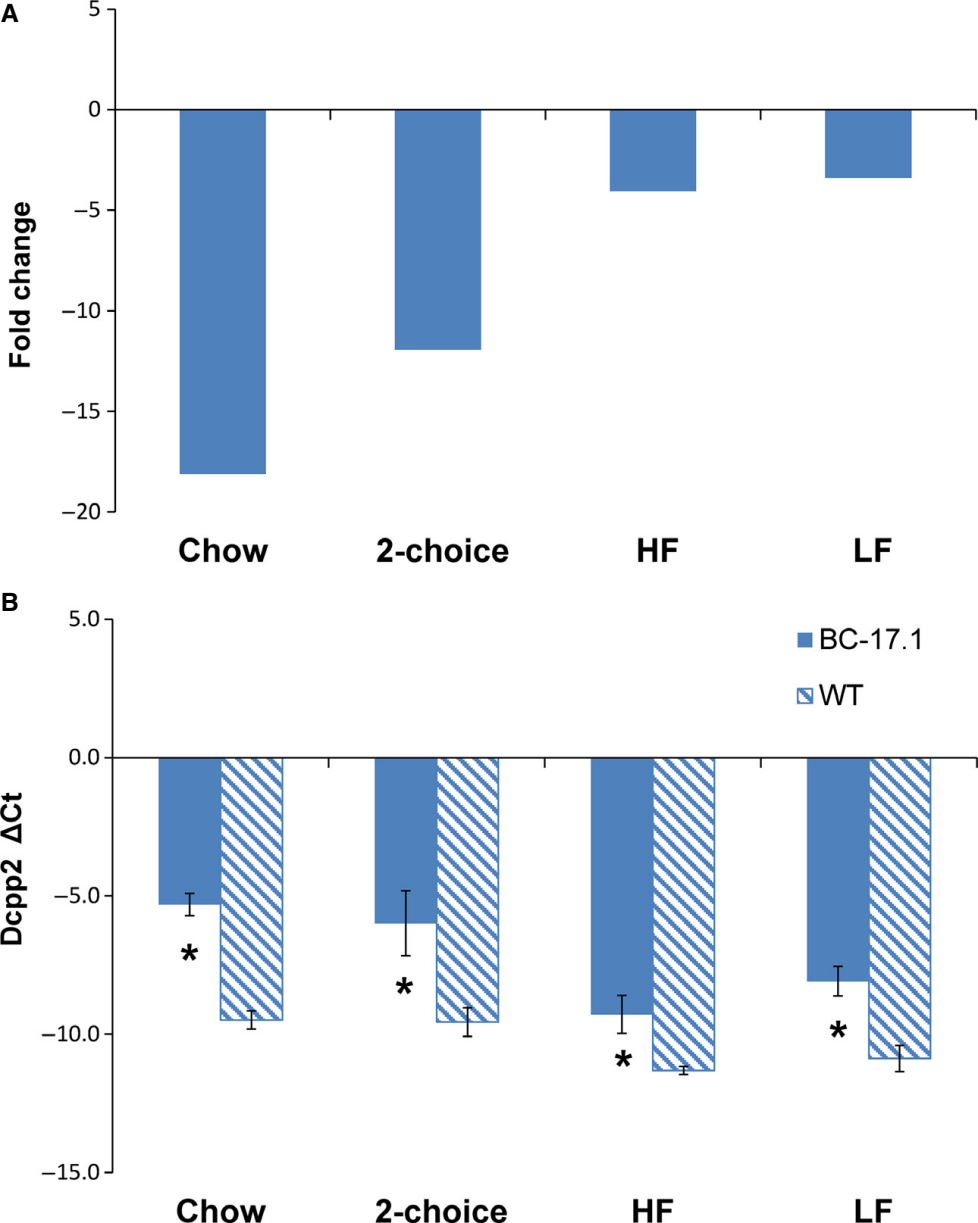
Effect of genotype on *Dcpp2* expression in sublingual gland (Δ*C*_T_ and FC) in four diet conditions. For each experimental group, gene expression levels were ascertained by quantitative real-time PCR (see Tables5, 6). (A) From the Δ*C*_T_ values, fold change in expression level was determined relative to the expression level in wild-type (WT) B6. (B) Bar diagrams depict means and standard deviations. Statistical significance was calculated based on the Δ*C*_T_ values. 2-choice refers to the fat- versus carbohydrate-rich diets. *P < 0.01. *n* = 6–14.

Our qRT-PCR analysis of *Dcpp2* expression revealed a main effect of genotype in mice fed either standard rodent chow (*P* < 0.0001) or the 2-choice macronutrient selection diet (*P* < 0.001) (Table4). *Dcpp2* expression was decreased by −18-fold in chow-fed B6.CAST-17.1 subcongenic mice compared to wild-type B6 mice (Fig.3A). The Δ*C*_T_ value for *Dcpp2* in subcongenic mice was −5.3 ± 0.4 compared to −9.5 ± 0.3 in controls (*P *< 0.001) (Table5). In animals selecting from macronutrient diets, that is, choosing between carbohydrate/protein versus fat/protein, *Dcpp2* expression was again significantly decreased in carbohydrate-preferring B6.CAST-17.1 subcongenic mice by −11.9-fold compared to fat-preferring wild-type mice (Fig.3A). Normalized to the geometric mean of the reference genes, the mean Δ*C*_T_ values were −5.9 ± 1.1 versus −9.5 ± 0.5, respectively (*P *< 0.001) (Fig.3B).

**Table 4:** Analysis of variance results for Δ*C*_T_ values

Gene symbol	Gland	Diet	Genotype effect	Post hoc comparison^1^ *P*-value	Fold change CON vs. WT	Diet effect	Post hoc comparison^1^ *P*-value	Fold change LF vs. HF; 2-choice vs. chow	Genotype × Diet
Dcpp2^2^	SL	Chow	*F* = 326.24 *P* < 0.0001	*P* < 0.001	−18.1	F = 3.10 *P* = 0.08	–	–	ns
	SL	2-choice		*P* < 0.001	−11.9		–	–	
	SL	HF	*F* = 18.84 P = 0.0002	*P* < 0.05	−4.0	ns	–	–	ns
	SL	LF		*P* < 0.05	−6.9		–	–	
Prrt1	SL	Chow	*F* = 4.53 *P* = 0.03	ns	–	ns	–	–	ns
	SL	2-choice		*P* < 0.05	1.6		–	–	
	SL	HF	ns	–	–	ns	–	–	ns
	SL	LF		–	–		–	–	
	SM	Chow	*F* = 13.86 *P* = 0.0006	ns	–	ns	–	–	ns
	SM	2-choice		*P* < 0.01	1.6		–	–	
	SM	HF	ns	–	–	ns	–	–	ns
	SM	LF		–	–		–	–	
	P	Chow	F = 3.56 P = 0.06	–	–	*F* = 65.17 *P* < 0.0001	CON, *P* < 0.001	−2.0	ns
	P	2-choice		–	–		WT, *P* < 0.001	−2.4	
	P	HF	Not tested	–	–	Not tested	–	–	Not tested
	P	LF		–	–		–	–	
Has1	SL	Chow	*F* = 8.65 *P* = 0.005	ns	1.6	ns	–	–	ns
	SL	2-choice		ns	1.5		–	–	
	SL	HF	ns	–	–	ns	–	–	ns
	SL	LF		–	–		–	–	
	SM	Chow	ns	–	–	ns	–	–	ns
	SM	2-choice		–	–		–	–	
	SM	HF	*F* = 38.09 *P* < 0.0001	*P* < 0.01	−2.4	*F* = 6.64 *P* < 0.05	WT, *P* < 0.05	1.9	*P* = 0.11
	SM	LF		*P* < 0.001	−4.1		CON, ns	–	
	P	Chow	ns	–	–	ns	–	–	ns
	P	2-choice		–	–		–	–	
	P	HF	Not tested	–	–	Not tested	–	–	Not tested
	P	LF		–	–		–	–	

1 Bonferroni. ns, non significant; CON, subcongenic; WT, wild type; HF, high-fat; LF, low-fat.

2 *Dcpp2* showed very low abundance in SM and P. 2-choice indicates fat- versus carbohydrate-rich diets. SL, sublingual; SM, submandibular; P, parotid.

**Table 5:** Gene expression (Δ*C*_T_) in salivary glands of B6.CAST-17.1 homozygous subcongenic compared to wild-type mice, when fed chow or 2-choice macronutrient selection diet

Gene symbol	Wild-type	Subcongenic		Wild-type	Subcongenic	
Tissue	#5001 Chow		*P*-value	2-choice macronutrient diet		*P*-value
*Dcpp2*						
Sublingual	−9.49 ± 0.33	−5.31 ± 0.40	<0.001	−9.56 ± 0.52	−5.99 ± 1.18	<0.001
*Prrt1*						
Sublingual	8.36 ± 0.40	8.11 ± 0.47	ns	8.63 ± 0.97	7.91 ± 0.94	<0.05
Submandibular	7.03 ± 0.36	6.55 ± 0.49	ns	7.38 ± 0.73	6.68 ± 0.47	<0.01
Parotid	9.07 ± 0.50	8.95 ± 0.52	ns	10.33 ± 0.34	9.92 ± 0.44	ns
*Has1*						
Sublingual	9.26 ± 0.36	8.58 ± 1.00	ns	9.32 ± 0.78	8.74 ± 0.70	ns
Submandibular	6.72 ± 0.65	6.24 ± 0.72	ns	5.94 ± 1.18	6.30 ± 0.88	ns
Parotid	8.60 ± 0.90	8.88 ± 0.44	ns	8.88 ± 0.44	8.87 ± 0.49	ns

Multiple reference genes were used to normalize the qRT-PCR data (Vandesompele et al. 2002). Genotype comparisons were performed using individual Δ*C*_T_ values. Values are mean ± SD for 8–11 animals per group. *P*-values were obtained using the Student's *t*-test with Bonferroni correction, ns, non significant.

To test whether carbohydrate consumption increases *Dcpp2* expression in the subcongenic strain, and to control for the effects of variation in self-selected macronutrient intake, we conducted another experiment in which diet-naïve animals of both genotypes were fed a commercial diet containing either 58% kcal or 11% kcal from fat. The results showed a main effect of genotype [*F*(1, 24) = 18.84, *P* < 0.001] but not of diet [*F*(1, 24) = 0.47, *P *= 0.49] (Table4). Specifically, sublingual *Dcpp2* expression was lower in B6.CAST-17.1 subcongenic mice, when fed either a high- or low-carbohydrate diet, by −4.0- and −6.9-fold, respectively, compared to controls. The mean Δ*C*_T_ value was −9.3 ± 0.7 for subcongenic mice fed HF diet compared to −11.3 ± 0.1 for the controls (*P *< 0.001) (Table6). Significantly decreased expression was also observed in subcongenic mice fed LF diet, that is, the mean Δ*C*_T_ value was −8.1 ± 0.5 compared to −10.9 ± 0.5 for the controls (*P *< 0.01) (Table6; Fig.3B). Our data indicated very low-level, highly variable expression of *Dcpp2* in both the parotid and submandibular glands (data not shown).

**Table 6:** Gene expression (Δ*C*_T_) in salivary glands of B6.CAST-17.1 homozygous subcongenic compared to wild-type mice, when fed high- or low-fat diet

Gene symbol	Wild-type	Subcongenic		Wild-type	Subcongenic	
Tissue	High-fat^1^		*P*-value	Low-fat^1^		*P*-value
*Dcpp2*						
Sublingual	−11.31 ± 0.14	−9.29 ± 0.69	<0.05	−10.88 ± 0.47	−8.09 ± 0.53	<0.05
*Prrt1*						
Sublingual	7.24 ± 0.42	6.52 ± 0.33	ns	7.18 ± 0.56	7.16 ± 0.86	ns
Submandibular	7.67 ± 0.62	7.70 ± 0.35	ns	7.95 ± 0.93	7.84 ± 0.77	ns
*Has1*						
Sublingual	7.37 ± 0.46	6.24 ± 1.03	ns	7.21 ± 0.61	7.66 ± 0.45	ns
Submandibular	5.83 ± 0.91	7.09 ± 0.73	<0.01	4.93 ± 0.81	6.77 ± 0.71	<0.001

1 High-fat, D12331; low-fat D12329 (Research Diets, Inc.). The qRT-PCR data were normalized using multiple reference genes (Vandesompele et al. 2002). Genotype comparisons were performed using individual Δ*C*_T_ values. Values are mean ± SD for 5–11 animals per group. *P*-values were obtained using the Student's *t*-test with Bonferroni correction, ns, nonsignificant.

Thus, with each diet tested, *Dcpp2* expression in the sublingual gland was decreased in subcongenic mice compared to wild-type controls. *Dcpp2* mRNA was expressed in gland-specific manner, as previously indicated (Mullins et al. 2006).

### *Prrt1* expression in the salivary glands was altered by genotype and diet

**Figure 4:**
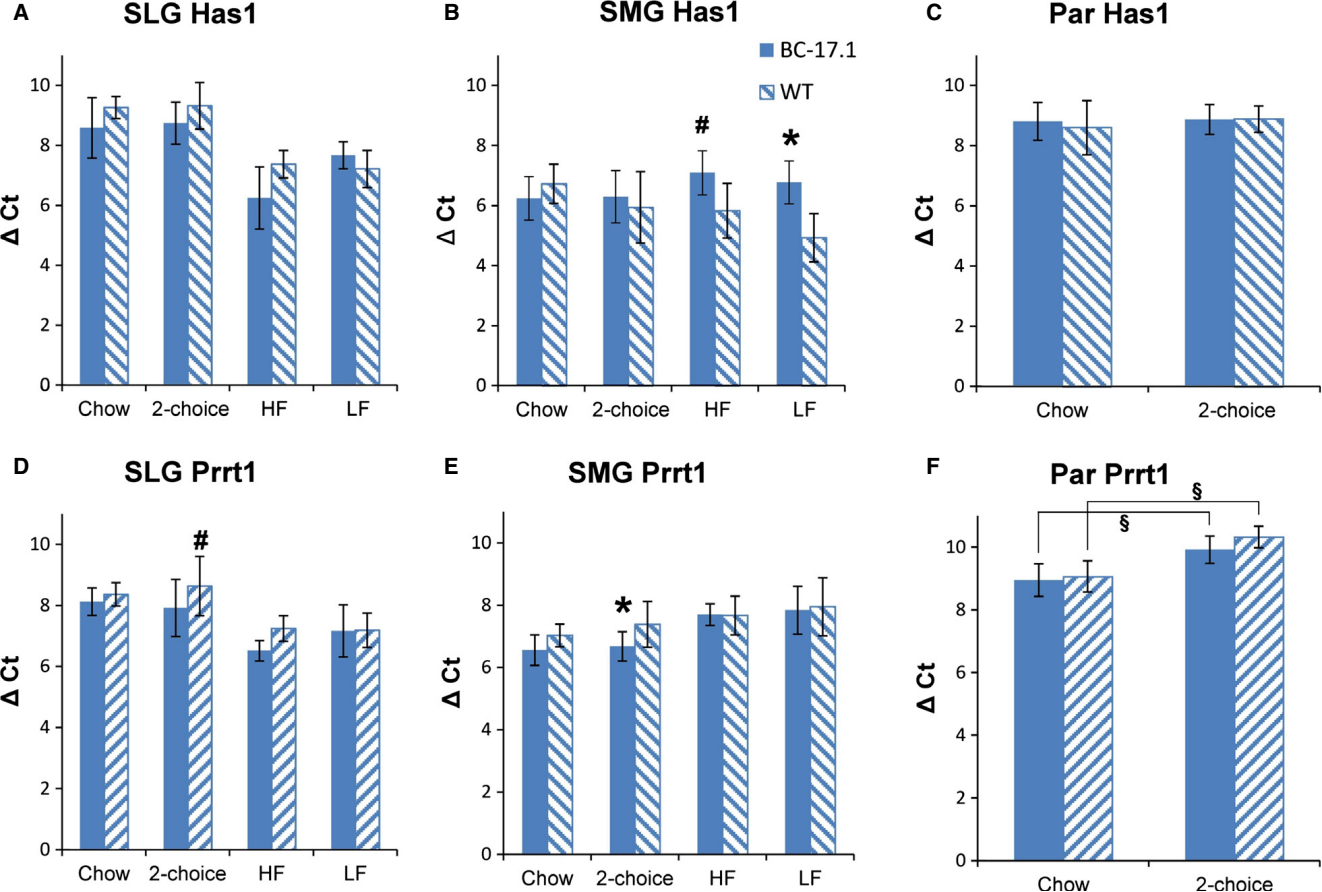
Effect of genotype on *Prrt1* and *Has1* expression (Δ*C*_T_) in sublingual, submandibular, and parotid glands. For each experimental group, gene expression levels were determined by quantitative real-time PCR (see Tables5, 6). Statistical comparisons were made using the Δ*C*_T_ values. Bar diagrams depict means and standard deviations. 2-choice indicates a choice between fat- and carbohydrate-rich diets. Genotype comparisons; *P < 0.01, #*P* < 0.05. Diet comparisons are illustrated by horizontal bars in F; §*P* < 0.001. *n* = 5–11.

We measured *Prrt1*expression in the salivary glands of subcongenic and wild-type mice under these diet conditions: (1) rodent chow ad libitum, (2) macronutrient selection diet for 2 days, and (3) high- or (4) low-fat diet for 2 days (Fig.4). With the chow and macronutrient selection diets, genotype affected *Prrt1* expression in both the sublingual (*P* < 0.05) and submandibular (*P* < 0.001) glands, whereas the effect in parotid was not significant (*P* = 0.06) (Table4). *Prrt1* expression, normalized to the geometric mean expression of two reference genes, was significantly increased in the sublingual gland of subcongenic mice compared to wild-type controls when the animals were fed macronutrient selection diets (1.6-fold, *P < *0.05) but not when fed chow (1.1-fold) (Fig.4D). In the submandibular gland, subcongenic mice also showed higher *Prrt1* expression (1.6-fold, *P *< 0.01) but not when fed chow (Fig.4E). Overall, diet strongly affected *Prrt1* expression in the parotid gland, independent of genotype [*F*(1, 39 = 65.17, *P* < 0.0001] (Table4). That is, *Prrt1* expression was significantly lower in both subcongenic (−2.0-fold, *P* < 0.001) and wild-type (−2.4-fold, *P* < 0.001) mice fed macronutrient selection diet compared to chow (Table4). The effects of high- or low-fat diets on *Prrt1* expression were not examined in parotid gland.

In summary, *Prrt1* expression in both the sublingual and submandibular glands was increased by genotype. In the parotid gland, however, *Prrt1* expression was decreased in both mouse strains as an effect of diet. Thus, we observed a diet effect on *Prrt1* gene expression that was limited to the parotid gland.

### *Has1* expression in the salivary glands was altered by genotype and diet

We compared *Has1* expression in the salivary glands of subcongenic versus wild-type mice under different diet conditions: (1) rodent chow ad libitum, (2) macronutrient selection diet for 2 days, and (3) high- or (4) low-fat diet for 2 days (Fig.4). *Has1* expression overall was increased by ≥1.5-fold in subcongenic versus wild-type mice in the sublingual [genotype, *F*(1, 45) = 8.65, *P* < 0.01], but not submandibular or parotid glands, when the mice had a choice of macronutrient diets as compared to chow feeding (Table4; Fig.4B and C). Genotype differences were not detected in post tests. In contrast, with HF and LF diets, we found significant effects of both genotype [*F*(1, 32) = 38.09, *P* < 0.0001] and diet [*F*(1, 32) = 6.64, *P *< 0.01] on *Has1* expression in submandibular gland, but no interaction between the two factors was found [*F*(1, 32) = 2.63, *P* = 0.11] (Table4). *Has1* expression in the submandibular glands was significantly reduced in subcongenic mice compared to wild-type mice (Fig.4B), whether the animals were fed LF (−4.1-fold; *P* < 0.001) or HF (−2.4-fold; *P *< 0.01) diet. The mean Δ*C*_T_ values were 7.1 ± 0.7 for subcongenic mice fed HF diet compared to 5.8 ± 0.9 for the controls (*P *< 0.01); and when fed LF diet, 6.8 ± 0.7 for subcongenic mice compared to 4.9 ± 0.8 for the controls (*P *< 0.001) (Table6). The effects of HF or LF diets on *Has1* expression in parotid gland were not examined.

Thus, *Has1* expression was increased by genotype in the sublingual gland of animals fed with macronutrient diets or chow (Tables4, 5). In submandibular gland, *Has1* expression was decreased by genotype in animals fed LF or HF diet and this reduction was more pronounced on the LF diet (Table4). However, the interaction between genotype and diet was not significant.

### Hyaluronic acid level in saliva was positively correlated with proportional fat intake with macronutrient selection diets, independent of genotype

**Figure 5:**
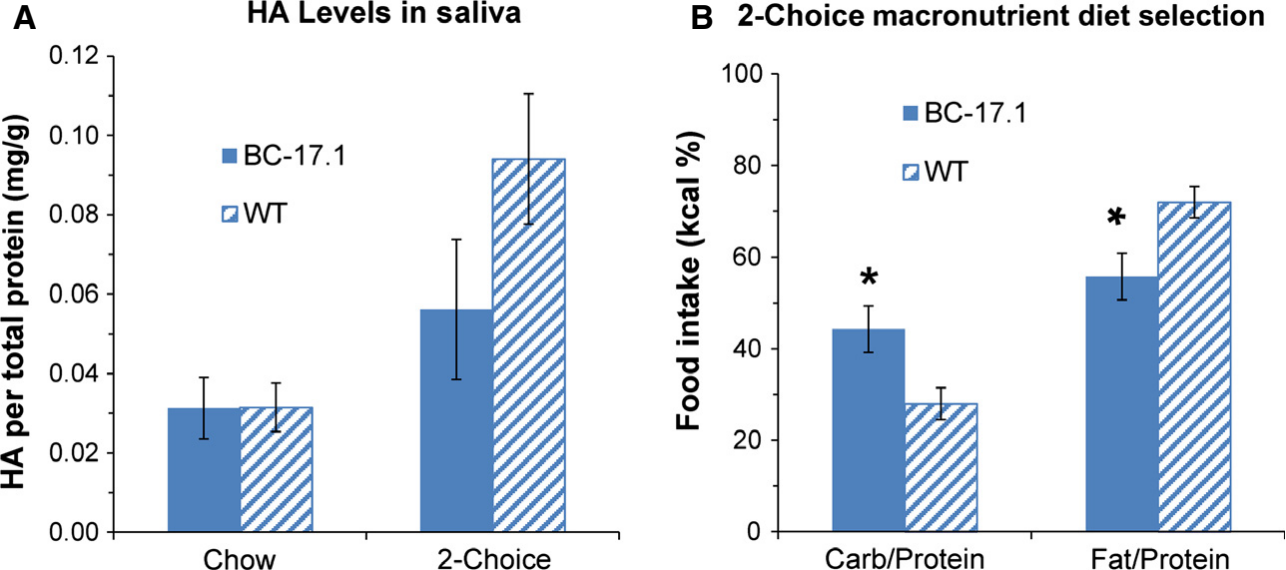
Effect of Chow versus macronutrient selection diet on salivary hyaluronic acid (HA) level. The animals were fed macronutrient selection diet for 2 day and then saliva samples were collected from subcongenic (*n* = 9) and wild-type (*n* = 9) mice. Bar diagrams depict means and standard errors. (A) Differences between genotype group means were not statistically significant. (B) Subcongenic mice selected/consumed proportionately more kcal from the carbohydrate-rich diet (*P* < 0.05), whereas wild-type mice showed a preference for the fat-rich diet (*P* < 0.05).

**Figure 6:**
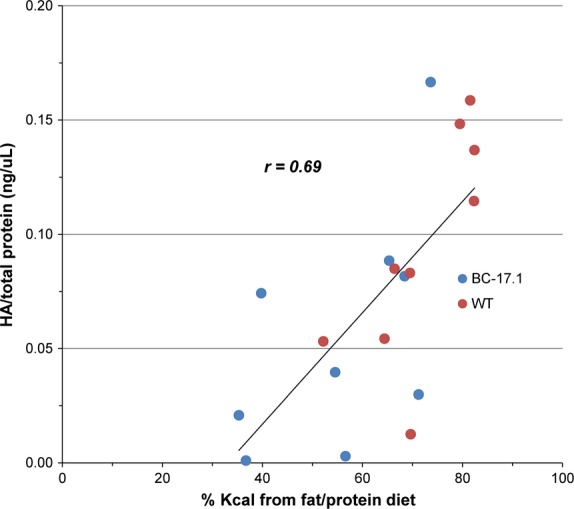
Level of hyaluronic acid in saliva was positively correlated with the proportion of kcal selected from the fat/protein diet. The proportion of total kcal consumed from fat/protein (vs. carbohydrate/protein) in this macronutrient diet choice paradigm was positively correlated with hyaluronic acid (HA)/total protein in mouse saliva (*r* = 0.69, *P* < 0.0001). *n* = 9 per genotype.

*Has1* encodes hyaluronan synthase, a plasma membrane enzyme that produces hyaluronan or hyaluronic acid, an essential component of the extracellular matrix (Abe and Dawes 1978). Hyaluronan is a large glycosaminoglycan which has been reported in human saliva (Pogrel et al. 1996) and whose physical properties include lubrication (Park et al. 2010). Our studies found differences in *Has1* expression in mouse salivary glands, as an effect of both genotype and diet. We therefore evaluated diet-induced changes in the HA content of saliva obtained from subcongenic and wild-type mice, when the animals were fed standard rodent chow, and then again after they were switched to the macronutrient selection diets for 2 days. Overall, salivary HA levels, adjusted for total protein, were altered by diet [*F*(1, 32) = 11.28, *P* = 0.002] but not by genotype [*F*(1, 32 = 2.14, *P* = 0.15] (Fig.5). With chow feeding, salivary HA level was equivalent between the two genotypes [subcongenic (*n* = 9): 0.031 ± 0.02 ng/µL vs. wild-type (*n* = 9): 0.031 ± 0.02 ng/µL, *P *= 0.99] (Fig.5A). After 2 days of macronutrient selection diet, we again observed no difference in salivary HA levels between the two genotypes [subcongenic (*n* = 9): 0.056 ± 0.05 vs. wild-type (*n* = 9): 0.094 ± 0.05, *P* = 0.19], despite strain differences in self-selected intake from the fat- and carbohydrate-rich diets (Fig.5A–B). The diet effect was due to a higher level of HA in the saliva of fat-preferring wild-type animals fed macronutrient selection diets, compared with chow [*t* = 3.40, adj*P* = 0.007, Bonferroni]. In contrast, carbohydrate-preferring subcongenic mice showed no difference in HA level when fed macronutrient selection, compared with chow diet [*t* = 1.35, adj*P* = 0.75]. We then asked whether the animals’ preference for a macronutrient-rich diet (Kumar et al. 2010) affected HA levels in saliva. We detected a significant and positive correlation between proportional fat intake (percent by energy) and salivary HA level (Pearson *r* = 0.69, *P *= 0.0010) (Fig.6). To apply statistical control for variation in the animals' self-selected intake of fat- or carbohydrate-rich diets, we performed an analysis of covariance (ANCOVA). These results demonstrated a significant effect of proportional fat intake (percent by energy) on salivary HA level [*F*(1, 15) = 16.44, *P* = 0.001] that was independent of strain [*F*(1, 15) = 0.00, *P* = 0.98].

In summary, we observed no genotype effects on salivary hyaluronic acid level following exposure to chow feeding or macronutrient selection. However, the macronutrient selection diets, overall, modified the hyaluronic acid content of saliva, an effect that was due to higher levels of HA in fat-preferring wild-type mice. Further analysis revealed a positive association between the concentration of hyaluronan in saliva and proportional fat intake in mice. Therefore, a higher consumption of fat (by energy) resulted in increased salivary hyaluronan content.

### Hyaluronic acid level in saliva was altered by genotype but not by high- or low-fat diets

**Figure 7:**
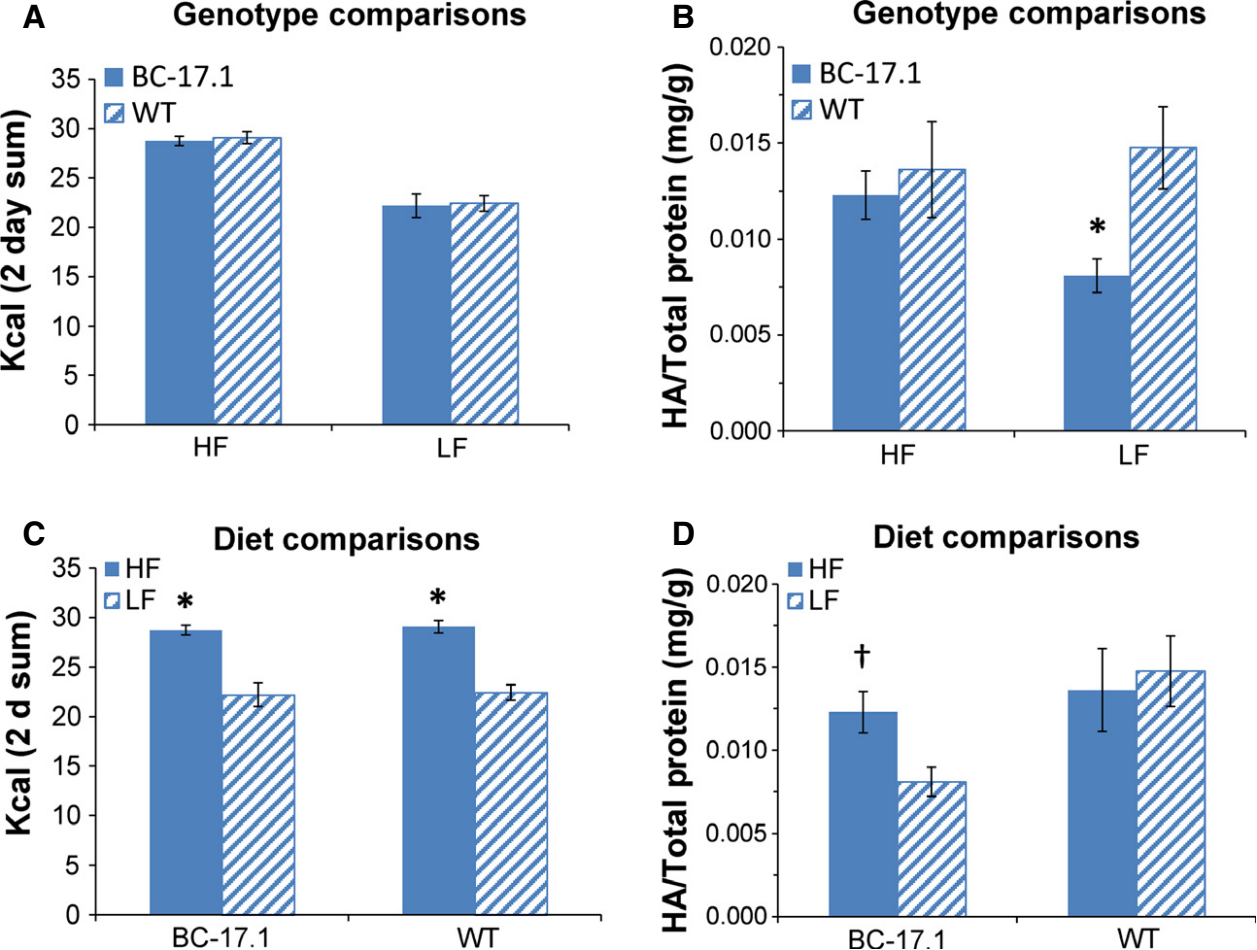
Effects of genotype and diet (high- or low-fat) on hyaluronic acid level in saliva. The animals were fed either high- (HF) or low-fat (LF) diet for 2 days and then saliva samples were collected from subcongenic (*n* = 10) and wild-type (*n* = 10) mice. Bar diagrams depict means and standard errors. (A) On HF or LF diet, subcongenic and wild-type mice consumed equivalent amounts of kcal. (B) LF-fed, subcongenic mice had lower HA levels (*P* < 0.05, vs. wild-type controls). (C, D) Re-plotted data from A and B to facilitate comparison by diet. Both subcongenic and wild-type mice ate more HF diet (*P* < 0.0001, vs. LF diet) (C). (D) Subcongenic mice fed HF diet had higher HA levels (†*P *= 0.019, vs. LF, uncorrected *t*-test).

Next, we examined the level of HA enzyme in saliva collected from subcongenic and wild-type mice after 2 day exposure to either high- or low-fat diet. There were no genotype differences in the intake of HF or LF diets (Fig.7A). However, we observed a significant overall effect of genotype [*F*(1, 35) = 10.14, *P* < 0.05] but not diet [*F*(1, 35) = 1.48, *P* = NS] (Fig.7) on salivary HA. Salivary HA was lower in subcongenic mice fed LF diet (*P* < 0.05, vs. controls) (Fig.7B), despite similar amounts of food intake. Both strains consumed more HF than LF diet (*P* < 0.0001) (Fig.7C). Overall, there was no effect of diet on salivary HA levels [*F*(1, 35) = 1.48, *P* = 0.43] (Fig.7D). Nevertheless, subcongenic mice fed HF diet had higher HA levels (*P *= 0.019, vs. LF, uncorrected *t*-test). Also, we found no correlations between mRNA levels (Δ*C*_T_) and HA/total protein or between fat intake and HA level in HF- and LF-fed animals (data not shown). Thus, we found no evidence for an association between salivary HA and fat intake in this experiment.

## Discussion

We investigated *Dcpp2*, *Prrt1,* and *Has1* as plausible gene candidates for the carbohydrate selection/intake QTL *Mnic1*, based on their genetic positions within its genetic boundaries and expression in salivary glands, based on the important role of saliva in oral food processing and taste. Our studies found genotype differences in the expression of all three genes, in at least one major salivary gland, as well as diet effects on the expression of *Prrt1* and *Has1* in different glands. We also discovered an association between diet composition and hyaluronan content in mouse saliva.

Originally, we hypothesized that a genotype-by-diet interaction affecting gene expression would reveal a potentially causal gene, but it is warranted to reevaluate this assumption in light of the lack of such support by results for the three candidates tested. The postulate of a statistically significant interaction between genotype and a trait-related factor (e.g., diet) on gene expression as a prerequisite for identifying candidate quantitative trait gene(s) is not widely shared in the current literature. Instead, it has been suggested that differentially expressed genes regulated in *cis*, that is, by a genetic polymorphism(s) located nearby on the chromosome, may be regarded as likely candidates (1) if they are positioned under the phenotypic QTL and (2) their expression is correlated with the phenotype (Verdugo et al. 2010). Others have postulated that potentially causal genes (3) be expressed in a trait-relevant tissue, and (4) vary in expression level by different states related to the trait (Flint et al. 2005; Arbilly et al. 2006). The genes under consideration in our study fulfill the above criteria. *Dcpp2*, *Prrt1,* and *Has1* are located under the phenotypic QTL for preferential carbohydrate intake and *Dcpp2* expression is correlated with this phenotype, that is, mRNA abundance is decreased by 12-fold in the sublingual gland of carbohydrate-preferring, subcongenic mice compared to fat-preferring wild-type controls (Fig.3; Table4). Moreover, *Dcpp2*, *Prrt1,* and *Has1* were differentially expressed between strains in the salivary gland (a trait-relevant tissue); and their expression was increased or decreased for various diet conditions (states related to the trait or phenotype). In addition, available SNP data show the presence of nonsynonymous SNPs located in the coding region and modifying the protein sequence of both *Dcpp2* and *Has1*. Thus, our findings provide support for all three genes as possible candidates for effects of the *Mnic1* QTL.

Our observations of a gland-specific pattern in *Dcpp2* expression are in agreement with the report that p20 (now formally defined as *Dcpp)* expression in mouse sublingual gland is several times greater than in the parotid gland, and is barely detectable in submandibular gland (Bekhor et al. 1994). The p20/Dcpp mRNA was localized to demilune cells of the sublingual gland and intercalated duct cells of the parotid gland (Bekhor et al. 1994). Recently, a family of three closely linked, demilune cell and parotid protein (*Dcpp*) genes on mouse chromosome 17 have been identified, as well as a putative human *Dcpp* ortholog (Mullins et al. 2006). The function of the DCPP2 protein is not yet known (Mullins et al. 2006). Due to its secreted nature and the presence of a putative JRL (jacalin-related lectin-like) domain in both the mouse and human orthologs, it has been suggested that DCPP may be a lectin carbohydrate-binding protein or glycoprotein (Mullins et al. 2006). The sublingual gland secretions are largely mucous and contain complex carbohydrates that are attached to mucin proteins, whereas the adult mouse submandibular and parotid glands produce predominantly serous secretions (Miletich 2010). Our results showed that *Dcpp2* was expressed primarily in the sublingual gland, and was decreased by 12- and 18-fold in subcongenic mice versus B6 controls, depending on diet. According to the view that oral stimuli can affect the composition of saliva, or that saliva influences taste perception, (Spielman 1990; Zolotukhin 2013), we hypothesized that DCPP in the saliva could influence preferences for fat- or carbohydrate-rich diets. Although we found strong effects of genotype, we did not detect differences in *Dcpp2* expression related to diet. Our results thus provide evidence for the genetic, but not nutritional control of *Dcpp2* expression, in this animal model.

Effects of the macronutrient diets on *Prrt1* expression, as compared to chow, were confined to the parotid gland. In this gland, serous cells containing protein-storing granules are associated with the release of water and enzymes (Elkstrom et al. 2012) for example, amylase, that moisten and digest food (Miletich 2010). The salivary gland cell type associated with *Prrt1* is not known and information about its specific functions in saliva or any other tissue is not yet available. However, *Prrt1* encodes a transmembrane protein, whose function in general is to enable cells to respond and interact with their environment. A possible interaction of the PRRT1 protein with oral food stimuli remains to be determined. In response to a reviewer's query, we found no literature to support that the gene product of *Prrt1* has enzymatic activity; only a gene ontology annotation for “response to biotic stimulus” (www.informatics.jax.org/go).

To our knowledge, evidence for the expression of *Has1* mRNA in salivary glands is limited to a report by Terpe et al. (Terpe et al. 1994), who analyzed the tissue distribution of HA synthetase in human epithelial tissues using immuno-histochemical staining. In salivary gland (type not specified), strong staining for HA synthetase was demonstrated for acinus cells, the intercalated portion and ducts (Terpe et al. 1994), consistent with the secretion of fluid and enzymes (Elkstrom et al. 2012). Additional studies will be needed to determine the distribution of *Has1* expression among salivary gland tissues.

We are aware that the presence of regulatory SNPs, for example, in promoter regions, could affect mRNA abundance and thus be responsible for the observed expression variation in the genes of interest. However, studies of the mechanisms controlling transcription of a gene require the identification and characterization of its regulatory elements, which is beyond the scope of this study.

*Has1* encodes hyaluronan synthase which is one of three isoenzymes (HAS 1-3) that synthesize hyaluronan. Hyaluronan, also known as hyaluronate or hyaluronic acid (HA), is a glycosaminoglycan, a high molecular weight polysaccharide composed of alternating sequences of D-glucoronic acid and N-acetyl-D-glucosamine. HA is produced by fibroblasts and other connective tissue cells and is a major component of the extracellular matrix (Laurent and Fraser 1992; Tammi et al. 2011), where it can affect water transport. It is synthesized at the plasma membrane by *Has1* and then is extruded into the extracellular space where its functions include providing space between cells, lubrication of joints, and facilitating cell migration (Girish and Kemparaju 2007). HA is widely distributed throughout the body, including in the plasma and synovial fluid. There have been only a few investigations of the occurrence and possible role of hyaluronic acid in saliva (Pogrel et al. 1996, 2003; Tishler et al. 1998; Higuchi et al. 2009), for example, as a marker of dry mouth in human patients (Higuchi et al. 2009).

An association of *Has1* and hyaluronic acid with food intake has not been investigated previously. We report here a positive correlation between salivary HA levels and proportional fat intake, regardless of genotype. This result was unexpected, based on the well-known role of HA to act as a lubricant in many areas of the body. In particular, one might expect HA content in saliva to increase in response to a powdered, carbohydrate-rich diet, rather than an oily, fat-rich diet mixture. In an effort to explain this finding, we noted that our macronutrient selection diets contain cellulose fiber, also known as alphacel (Cat. No. 900453; MP Biomedicals, LLC; www.mpbio.com). This cellulose is derived from birch and maple tree pulp which contain tannins (www.ansci.cornell.edu/plants/toxicagents/tannin.html). It is known that hyaluronan levels result from a balance between biosynthesis by hyaluronan synthetase and enzymatic degradation, for example, by hyaluronidase (Tammi et al. 2011). Furthermore, it has been proposed that in foods, plant-derived phenolics and tannins may inhibit hyaluronidase activity (Bralley et al. 2008), thereby increasing the amount of hyaluronan present (Pogrel et al. 2003; Tammi et al. 2011) in the saliva. The amount of alphacel used in the macronutrient-rich diets was adjusted to energy density; thus the fat/protein diet mixture used in this study contains ∼35% more cellulose by weight than the carbohydrate/protein preparation does. We speculate that the higher alphacel content in the fat-rich diet is associated with greater tannin content, and this could be responsible for increasing the HA level in saliva in a positive fashion relative to fat intake. Recent studies on changes in salivary protein composition in response to diet, including tannins (Bennick 2002; Lamy et al. 2010) suggest that this proposition is worthy of further investigation. Consistent with this notion, salivary HA levels were not correlated with fat intake in mice eating the HF or LF diets, which do not contain any cellulose.

To date, we know of only one report describing the effects of food intake on hyaluronic acid, also known as hyaluronan, when it was measured in plasma for the purpose of optimizing a clinical test (Fraser and Gibson 2005). Test meal ingestion induced significant increases in plasma HA that were 1–13 times the fasting level in human subjects, presumably by displacement of hyaluronan from gastrointestinal tissues (Fraser and Gibson 2005). No difference was found in the plasma hyaluronan response to either high- or low-fat meals, or to glucose solution alone. It remains to be determined whether or not eating low- or high-fat food can produce differences in the hyaluronan content of saliva.

Afferent stimuli for the secretion of saliva include the act of eating. Food in the oral cavity activates a variety of receptors consisting of gustatory receptors, nociceptors, and olfactory receptors, as well as mechanoreceptors that are stimulated by chewing, which in turn activate sympathetic and parasympathetic autonomic nerves (Elkstrom et al. 2012), leading to stimulated salivary secretion. Only the minor glands secrete saliva spontaneously (in humans), for example, the von Ebner glands. Saliva then mixes with food stimuli, and the products of their interactions are perceived as taste and flavor sensations (Spielman 1990; Neyraud 2014). Saliva contains a very large number of unidentified and identified proteins that can be assigned to three main categories: enzymatic conversion, binding, and transport of substrates (Neyraud 2014). The enzymatic activities of saliva include amylase (complex carbohydrates), protease (proteins), and lipase (lipids). However, little is known about the interactions of salivary enzymes with diets or individual macronutrients and whether or not enzymatic actions alter the perception of nutrients (Neyraud 2014) and ultimately food choice. These questions require further study and should take into account the documented between-subject variability in saliva characteristics (Neyraud et al. 2012).

In summary, this study demonstrates that the genetic locus *Mnic1*affects the salivary gland expression of *Dcpp2* (sublingual), *Prrt1* (submandibular, sublingual), and *Has1* (submandibular), irrespective of diet. Unrelated to genotype, the type of diet clearly affected *Prrt1* expression (parotid). Further studies are needed to determine whether altered *Prrt1*expression in the parotid, or *Has1* expression in submandibular gland, is a cause or consequence of macronutrient-specific diet selection. Notably, our results suggest that the salivary hyaluronan content is affected by diet composition. This observation needs to be explored further by testing diets that contain varying levels of cellulose, as well as by manipulating the type and content of dietary fat.
